# Supplementary material for: YouTube as a Source of Patient Information Regarding Exercises and Compensated Maneuvers for Dysphagia
Source: Healthcare (Basel). 2021 Aug 23;9(8):1084. doi: 10.3390/healthcare9081084 (PMC8392155; doi:10.3390/healthcare9081084)
Supplement: Supplementary file 1 [file healthcare-09-01084-s001.zip › healthcare-1317471-supplementary.pdf]

Supplementary Table S1. Global quality scale

1. Poor quality, poor flow, most information missing, not helpful for patients
2. Generally poor, some information given but of limited use to patients
3. Moderate quality, some important information is adequately discussed
4. Good quality good flow, most relevant information is covered, useful for patients
5. Excellent quality and excellent flow, very useful for patients

Supplementary Table S2. DISCERN reliability tool

1. Is the video clear, concise, and understandable?
2. Are valid sources cited? (from valid studies, physiatrists, or physicians)
3. Is the information provided balanced and unbiased?
4. Are additional sources of information listed for patient reference?
5. Does the video address areas of controversy/uncertainty?
